# Functionalized cellulose nanofibrils in carbonate-substituted hydroxyapatite nanorod-based scaffold from long-spined sea urchin (*Diadema setosum*) shells reinforced with polyvinyl alcohol for alveolar bone tissue engineering

**DOI:** 10.1039/d3ra06165e

**Published:** 2023-11-03

**Authors:** Muhammad Amir Jamilludin, I Kadek Hariscandra Dinatha, Apri I Supii, Juliasih Partini, Dwi Liliek Kusindarta, Yusril Yusuf

**Affiliations:** a Department of Physics, Faculty of Mathematics and Natural Sciences, Universitas Gadjah Mada Yogyakarta 55281 Indonesia yusril@ugm.ac.id; b Research Centre for Marine and Land Bioindustry, National Research and Innovation Agency Lombok Utara 83352 Indonesia; c Department of Anatomy, Faculty of Veterinary Medicine, Universitas Gadjah Mada Yogyakarta 55281 Indonesia

## Abstract

In this study, carbonate-substituted hydroxyapatite (C-HAp) nanorods were synthesised using a dissolution-precipitation reaction on hydroxyapatite (HAp) nanorods based on long-spined sea urchin (*Diadema setosum*) shells. From the EDS analysis, the Ca/P molar ratio of C-HAp was 1.705, which was very close to the Ca/P of natural bone apatite of 1.71. The FTIR and XRD analyses revealed the AB-type CHAp of the C-HAp nanorods. The TEM showed the rod-like shape of nanosize C-HAp with a high aspect ratio. The antibacterial test against *Pseudomonas aeruginosa* and *Staphylococcus aureus* also showed that C-HAp had a high antibacterial activity. The C-HAp/PVA-based scaffolds were fabricated, using a freeze-drying method, for use in alveolar bone tissue engineering applications. There were various scaffolds, with no filler, with microcrystalline cellulose (MCC) filler, and with cellulose nanofibrils (CNF) filler. The physicochemical analysis showed that adding PVA and cellulose caused no chemical decomposition but decreased the scaffold crystallinity, and the lower crystallinity created more dislocations that can help cells proliferate well. The antibacterial activity showed that the CNF induced the higher antibacterial level of the scaffold. According to the SEM results, the micropores of the C-HAp/PVA/CNF can provide a place for cells to grow, and its porosity can promote cell nutrient supply. The macropores of the C-HAp/PVA/CNF were also suitable for cells and new blood vessels. Therefore, the C-HAp/PVA/CNF scaffold was examined for its cytocompatibility using the MTT assay against NIH/3T3 fibroblast cells with a 24 h incubation. The C-HAp/PVA/CNF scaffold showed a high cell viability of 90.36 ± 0.37% at a low scaffold dose of 31.25 μg mL^−1^. The scaffold could also facilitate NIH/3T3 cells to attach to its surface. The IC_50_ value had also been estimated to be 2732 μg mL^−1^.

## Abstract 2

The CNF were functionalized in C-HAp nanorods-based scaffold with PVA reinforcement to promote high cell viability and cell attachment for NIH/3T3 fibroblast cells, which potentially can be applied in alveolar bone tissue engineering.

## Graphical abstract

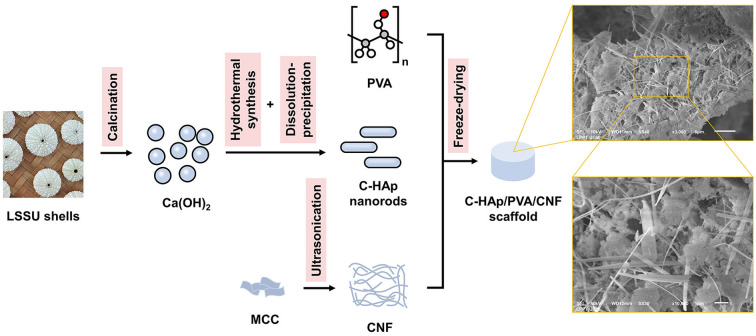

## 1. Introduction

Alveolar bone is part of the dental bone that can be affected by bone defects, bone loss, and periodontitis.^1^ To overcome these problems, a titanium bone implant is often placed in areas where bone resorption occurs.^2,3^ However, infection, implant failure, and reduced contact surface area are commonly found between the implant and the bone tissue, which can inhibit bone repair.^4^ Replacing implant materials with biomaterials is an excellent way to support bone regeneration.^5^ This is related to the required material properties for bone that must mimic the extracellular matrix (ECM) followed by biocompatibilitys and osteoconductive properties.^6^

Hydroxyapatite (HAp, Ca_5_(PO_4_)_3_OH) has been widely used as calcium phosphate for orthopaedic and dental applications because of its similarity to bone and tooth minerals.^7,8^ The HAp biomaterial can release calcium (Ca) and phosphate ions, which induce cell differentiation for new bone tissue formation.^9,10^ Human bone consists of rod-like shaped HAp with a nanosized length and diameter. The nanoscale size of HAp promotes greater cell adhesion. Because the HAp nanorods can mimic natural bone structure, they are more suitable for use in bone regeneration.^11^ The hydrothermal method is a simple method that can be used for HAp nanorod synthesis. The hydrothermal process induces nanocrystalline growth that strongly depends on the dissolution rate of raw materials.^12,13^ The dissolution rate can be controlled by the reaction temperature which influences the nanorod size.^14^ Therefore, the HAp nanorods can be designed hydrothermally to mimic the bone apatite structure at appropriate temperatures.

The HAp nanorods can be synthesised using synthetic or natural compounds. Natural compounds are preferred due to their essential trace elements, including iron (Fe), magnesium (Mg), zinc (Zn), and other inorganic elements, which can enhance the properties of HAp. The biogenic materials can be derived from marine shells, such as abalone mussel shells, green mussel shells, oyster shells, and sand lobster shells, all with high levels of Ca.^15–18^ This study used long-spined sea urchin (LSSU, *Diadema setosum*) shells from Indonesia that had never been used for HAp nanorod synthesis. The Ca content of the shells under the effect of the calcination temperature has been studied in previous research and was found to be over 93%.^19^

Biological hard tissue naturally contains 65% carbonated hydroxyapatite (CHAp) with 2–8% carbonate content in the apatite crystal.^20,21^ The carbonate content has a strong relationship with the solubility of apatite under biological conditions.^22^ The higher content of carbonate ions in the CHAp lattice crystal causes its crystallinity to be lower, so the CHAp is more soluble than HAp.^23^ Hence, CHAp has a higher osteoconductivity that can support cell differentiation better than HAp.^24–27^ Some studies have investigated a dissolution–precipitation method to incorporate carbonate and phosphate ions into materials such as calcite or gypsum blocks using carbonate and phosphate solutions to obtain carbonate apatite.^28–31^ Therefore, in this study, the method firstly substituting carbonate ions into the HAp nanorod block to obtain C-HAp nanorods and then the effect of the dissolution–precipitation reaction on HAp in carbonate solution was determined.

As a bone apatite material, the C-HAp nanorods in powder form cannot be used directly as a bone substitute material due to the risk of inducing an inflammatory response upon *in vivo* implantation.^23^ The bone substitute material must be formed in a three-dimensional (3D) porous scaffold. The porous scaffold is expected to be biocompatible and biodegradable, so that it will support cell growth, differentiation, and proliferation for bone regeneration.^32^ The scaffold must contain pores, including macropores and micropores. The micropores must be smaller than 20 μm, which can be medium for cell growth and cell nutrient supply.^33^ The macropores used for cell distribution and new blood vessel formation had a size of approximately 100 μm.^34^ The pore area in the scaffold solid structure can be represented by porosity, which is also an important parameter for the determination of scaffold efficacy in bone regeneration.

Various methods have been used to fabricate scaffolds, such as electrospinning, gas foaming, and porogen leaching.^35–37^ In this study, a freeze-drying method was chosen for scaffold fabrication. The freeze-drying method is advantageous due to its simple lyophilisation process to produce a highly porous architecture scaffold under low temperature, and vacuum conditions,^38,39^ causing low contamination and no carbon reaction. The freeze-dried scaffold also had interconnected pores and a suitable porosity, which could promote bone regeneration.^40^ The freeze-drying method requires an appropriate polymer to act as a crosslinking agent to improve reinforcement in the scaffold structure, causing it to be suitable for bone tissue formation.^41^ In scaffold fabrication, the C-HAp nanorods can be reinforced with a biocompatible polymer, such as poly(vinyl alcohol) (PVA), which as a synthetic polymer is widely used for biomedical applications.^42,43^ The PVA can be physically crosslinked by lyophilising PVA solution without other material at a low temperature of about −30 °C,^44^ hence, the polymeric PVA has good potential for use in reinforcing the C-HAp nanorods to form a porous structure.

The most commonly used biopolymer in the biomedical field is cellulose due to its biocompatibility. The properties of cellulose can be improved by modifying its structure into nanocellulose. Nanocellulose includes cellulose nanocrystals (CNC) and cellulose nanofibrils (CNF), which are generally used as reinforcing fillers in composite materials.^45–48^ The CNF has a longer length than CNC with a fibril diameter similar to or larger than CNC, causing the CNF to have a higher aspect ratio and to exhibit a more significant reinforcement effect than CNC. However, CNF contains crystalline and amorphous cellulose, which means CNF is not as highly crystalline as CNC.^49^ CNF can be synthesised by using the ultrasonication treatment of microsize cellulose and involves no toxic chemicals. Because CNF has been reported as biomaterial with a low cytotoxicity effect,^50,51^ it has promise for use in novel tissue engineering applications. In this study, CNF was used as a filler in a C-HAp/PVA-based scaffold matrix.

The research reported here investigated CNF filler addition in a C-HAp/PVA matrix scaffold for alveolar bone tissue engineering. This work introduced the dissolution–precipitation method for synthesising C-HAp nanorods using a HAp nanorod block. Meanwhile, the HAp nanorods were prepared using a hydrothermal method, from LSSU shells which were used as a novel precursor for obtaining a Ca source. The C-HAp/PVA solution was made to fabricate a 3D scaffold used for mimicking bone structure. The CNF was also synthesised using an ultrasonication treatment on microcrystalline cellulose (MCC), which was used as filler in the C-HAp/PVA matrix to enhance the scaffold properties. These scaffolds were examined to determine their chemical, morphological, and antibacterial properties. The cell viability assay was also conducted to evaluate the cytocompatibility of the scaffold with the most potential for future use based on its properties.

## 2. Materials and methods

### 2.1. Materials

The LSSU shells used as the Ca source were collected from Buleleng, Bali, Indonesia. Diammonium hydrogen phosphate ((NH_4_)_2_HPO_4_) used as a phosphate source, and sodium hydrogen carbonate (NaHCO_3_) as a carbonate source were purchased from Merck (USA). The PVA with a molecular weight of 145 000 (100% hydrolysed) was purchased from Merck (Germany). The MCC was purchased from Sigma-Aldrich (USA) and used as the precursor for cellulose nanofibrils.

### 2.2. Methods

#### 2.2.1. Preparation of calcium hydroxide (Ca(OH)_2_) from LSSU shells

**Fig. 1:**
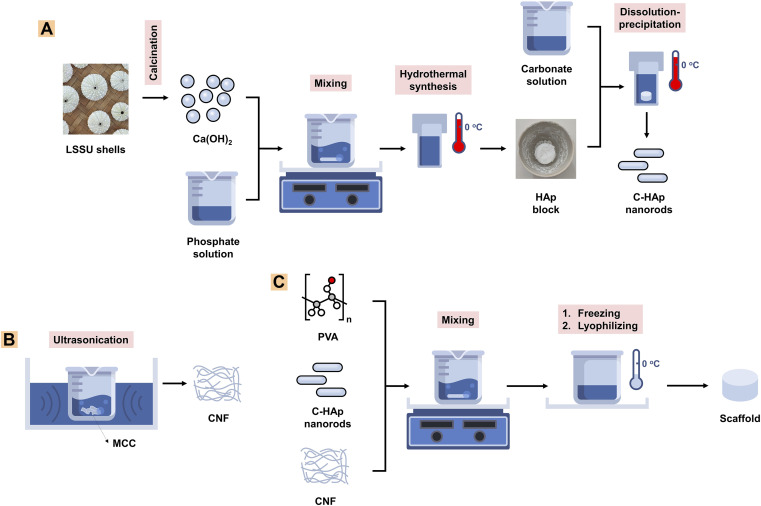
A schematic showing methods for (A) C-HAp nanorod synthesis, (B) CNF synthesis, and (C) scaffold fabrication.

The LSSU shells were boiled for 30 min to remove organic materials such as the shell meat and other impurities. The LSSU shells were then dried in an oven at 100 °C for 6 h. The cleaned LSSU shells were crushed and milled to obtain a powder with smaller particle sizes. The LSSU shell powder was characterised using Fourier transform infrared spectroscopy (FTIR) and X-ray diffraction (XRD). The powder was then calcined in furnace at 1000 °C for 6 h to obtain the CaO, powder (Fig. 1A). The powder was stored overnight in the furnace, as it was cooling down, which triggered the CaO to change into Ca(OH)_2_. The powder was then characterised to determine its chemical composition and structure using FTIR and XRD.

#### 2.2.2. Synthesis of carbonate-substituted hydroxyapatite (C-HAp) nanorods

The Ca(OH)_2_ (3.3 g) was added to 50 mL of distilled water and then stirred at a velocity of 350 rpm using a magnetic stirrer for 1 h at 37 °C. At the same time, 3.5323 g of (NH_4_)_2_HPO_4_ was added to 50 mL of distilled water and stirred with a stirring velocity of 350 rpm for 1 h at 37 °C. The phosphate solution was titrated dropwise into the calcium solution with drop rate of 1 mL min^−1^, and then stirred for 2 h at 60 °C to obtain a calcium–phosphate solution with a pH above 9. The calcium–phosphate solution was treated using a hydrothermal treatment. The Teflon vessel containing the solution was put in hydrothermal autoclave reactor and then heated in the oven at 200 °C for 24 h. The suspension was centrifuged with an angular velocity of 10 000 rpm for 30 min to obtain a precipitate which was the nano-HAp material. The precipitate was heated for 24 h at 60 °C to obtain the pure HAp nanorods. The HAp block was immersed in a 2 M NaHCO_3_ solution which was then treated hydrothermally for 24 h at 150 °C to obtain the C-HAp block. The block was washed three times using distilled water, and then dried for 24 h at 60 °C (Fig. 1A). The HAp and C-HAp blocks were then crushed into powder which was characterised using FTIR, XRD, and transmission electron microscopy (TEM). In particular, the C-HAp nanorod powder was characterised using a diffusion antibacterial test.

#### 2.2.3. Synthesis of cellulose nanofibrils (CNF)

An amount of the MCC (0.5 g) was dispersed in 40 mL of distilled water and stirred for 15 min at a velocity of 500 rpm, using a magnetic stirrer. The suspension was then sonicated using a water bath ultrasonicator for 60 min, at a frequency of 40 kHz, and kept at a low temperature (25 °C) to prevent excessive decomposition of the cellulose molecules. The suspension was centrifuged at an angular velocity of 10 000 for 30 min to obtain the precipitated CNFs. The precipitate was then lyophilised for 24 h to obtain CNF powder (Fig. 1B). The CNF powder was characterised using FTIR, XRD, and TEM.

#### 2.2.4. Fabrication of the C-HAp/PVA-based scaffolds

The C-HAp nanorods (0.9 g) were added to 9 mL of distilled water and stirred at 500 rpm for 1 h at 37 °C using a magnetic stirrer. Next, 1 g of PVA was dissolved in 10 mL of distilled water and stirred at 400 rpm for 2 h at 80 °C. The PVA solution (1 mL) was taken and added to the C-HAp solution. The mixture was stirred for 2 h at 60 °C, then stored overnight at −40 °C in a deep freezer before being lyophilised for 24 h at −20 °C. This sample was designated as C-HAp/PVA. The other samples were varied by adding cellulose powders, which were MCC and CNF. The cellulose powder (0.25 g) was dispersed in 5 mL of distilled water, and then poured into C-HAp/PVA solution and stirred for 1 h at 60 °C before being stored in the deep freezer (Fig. 1C). These samples were designated as C-HAp/PVA/MCC and C-HAp/PVA/CNF. The three scaffolds were characterised to determine their physicochemical and *in vitro* properties.

### 2.3. Characterisation

#### 2.3.1. Chemical composition analysis

The chemical compositions of the LSSU shells before and after calcination were characterised using X-ray fluorescence spectrometer (XRF, Rigaku NEX QC+ QuantEZ). The fluorescence data were used to determine the levels of calcium, phosphor, and other inorganic impurities in the shells. The change in the atomic levels was also evaluated.

#### 2.3.2. Analysis of functional groups

The functional groups within the LSSU shells before and after calcination, HAp, C-HAp, MCC, CNF, and the scaffold were determined using FTIR (ThermoFisher, Nicolet iS 10, Japan). The sample was mixed with potassium bromide (KBr) and pressed into a pellet. The infrared spectra were observed in the range 400–4000 cm^−1^.

#### 2.3.3. Crystallography analysis

The XRD diffraction analysis (PANanalytical, Type X'Pert Pro, Japan) was used to determine the crystallographic properties of the LSSU shells by analysing HAp, C-HAp, MCC, CNF, and the scaffold, before and after calcination. The XRD data was recorded in the range of 2*θ* = 5–90° using Cu-Kα radiation with *λ* = 0.154 nm.

#### 2.3.4. Morphology analysis

The TEM (Jeol, JEM-1400, Japan) was used to observe the morphology of the HAp, C-HAp, and CNF. The particle size and nanofibril diameter were calculated using ImageJ software based on randomly selected particles and nanofibrils. The scaffold morphology was observed using scanning electron microscopy (SEM, Jeol JSM-6510LA-1400, Japan). The micropore size of the scaffold was also calculated using ImageJ. The SEM analysis was combined with the energy dispersive X-ray spectroscopy (EDS), which measured the calcium and phosphor contents in the HAp and the C-HAp nanorod powders. The measured atomic masses were used to calculate the molar ratio of Ca/P in HAp and C-HAp.

#### 2.3.5. Antibacterial activity analysis

The diffusion antibacterial test was conducted to measure the antibacterial activity of the C-HAp nanorods and scaffolds using the disk diffusion method against *Pseudomonas aeruginosa* (Gram-negative bacteria) and *Staphylococcus aureus* (Gram-positive bacteria). The turbidity of the bacterial suspension was compared to the McFarland Standard of 0.5, which was equal to 1.5 × 10^8^ colony forming units (CFU mL^−1^). A 10 cm Petri dish was prepared with nutrient agar, and the surface was inoculated with bacteria using a sterile swab. A cylindrically compacted sample with a diameter of 6 mm was placed in the agar. After 24 h of incubation, the zone of inhibition (ZOI) formed was observed, and then the diameter was measured horizontally and vertically using a Vernier caliper.

#### 2.3.6. Cell viability assay

The scaffold particles were dispersed in 50 mL of distilled water to give serially diluted scaffold concentrations of 2000, 1000, 500, 250, 125, 62.5, and 31.25 μg mL^−1^. The serial of concentration was used for the analysis of the half maximal inhibitory concentration (IC_50_) analysis. The scaffold solution was stirred with a velocity of 350 rpm at 80 °C until it became a homogeneous solution. Then, the solution was sonicated at 60 °C for 60 min and then stored in the refrigerator before cell culture and seeding.

Mouse fibroblast cells (NIH/3T3) were chosen for the cell study, because they could mimic the alveolar bone tissue. The NIH/3T3 cells were cultured in DMEM, High Glucose (Gibco, USA), 10% Bovine Calf Serum (Sigma-Aldrich, USA), 2% Penicillin–Streptomycin (Gibco, USA), and 0.5% Fungizone (Gibco, USA). The NIH/3T3 cells were seeded at the bottom of the 96-well plate with a density of 2 × 10^4^ cells per well. The cells were incubated at 37 °C in 5% CO_2_ for 24 h. Next, 100 μL of the scaffold solution was added to the cells in three wells for each serially diluted concentration, then incubated at 37 °C in 5% CO_2_ for 48 h.

The NIH/3T3 cell viability was assessed during the incubation time of 24 h using the MTT assay. The measurement was taken for the C-HAp/PVA/CNF scaffold with the best result for the physicochemical–antibacterial properties and the well without scaffold as control. In each well, the medium was removed slowly with a micropipette, and then 100 μL of MTT solution (Bio Basic, USA) with a concentration of 0.5 mg mL^−1^ was added to the well and incubated for 4 h. Then, dimethyl sulfoxide (DMSO) (Merck, Germany) was added to the well at a concentration of 100 μL per well. The absorbance was measured using a Tecan Spark® analyser (Tecan, Switzerland) at 570 nm. The cell viability was determined from the absorption value of the culture test, which referred to the percentage absorption for the unstimulated control culture. The cell viability was calculated with eqn (1):1
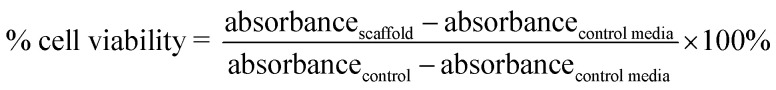


The IC_50_ was used to determine the maximum safe dose of the scaffold, which potentially inhibited the cell growth by 50%. This value was calculated using non-linear curve fitting.

#### 2.3.7. Statistical analysis

The cell viability and antibacterial activity data were determined using a one-way analysis of variance (ANOVA), and the results were presented as mean ± standard deviation (SD), followed by the Tukey method to compare the means tests and a *p*-value <0.05 was considered to be statistically significant.

## 3. Results and discussion

### 3.1. Properties of the raw materials

The LSSU shells were calcined before being used as Ca precursors in the HAp synthesis. The calcination process caused the CaCO_3_ content to decompose into CaO and CO_2_, followed by chemical reaction shown in eqn (2). The decomposition reaction occurred because of the heat energy received by atoms in the CaCO_3_ molecule structure, which triggered the atoms to move faster and break the chemical bonds of CaCO_3_.^19^ After the calcination process, because of the cooling of the temperature overnight, the CaO tended to react with the water molecules and became Ca(OH)_2_ (eqn (3)).

2 CaCO_3(s)_ → CaO_(s)_ + CO_2(g)_

3 CaO_(s)_ + H_2_O_(l)_ → Ca(OH)_2(s)_

**Fig. 2:**
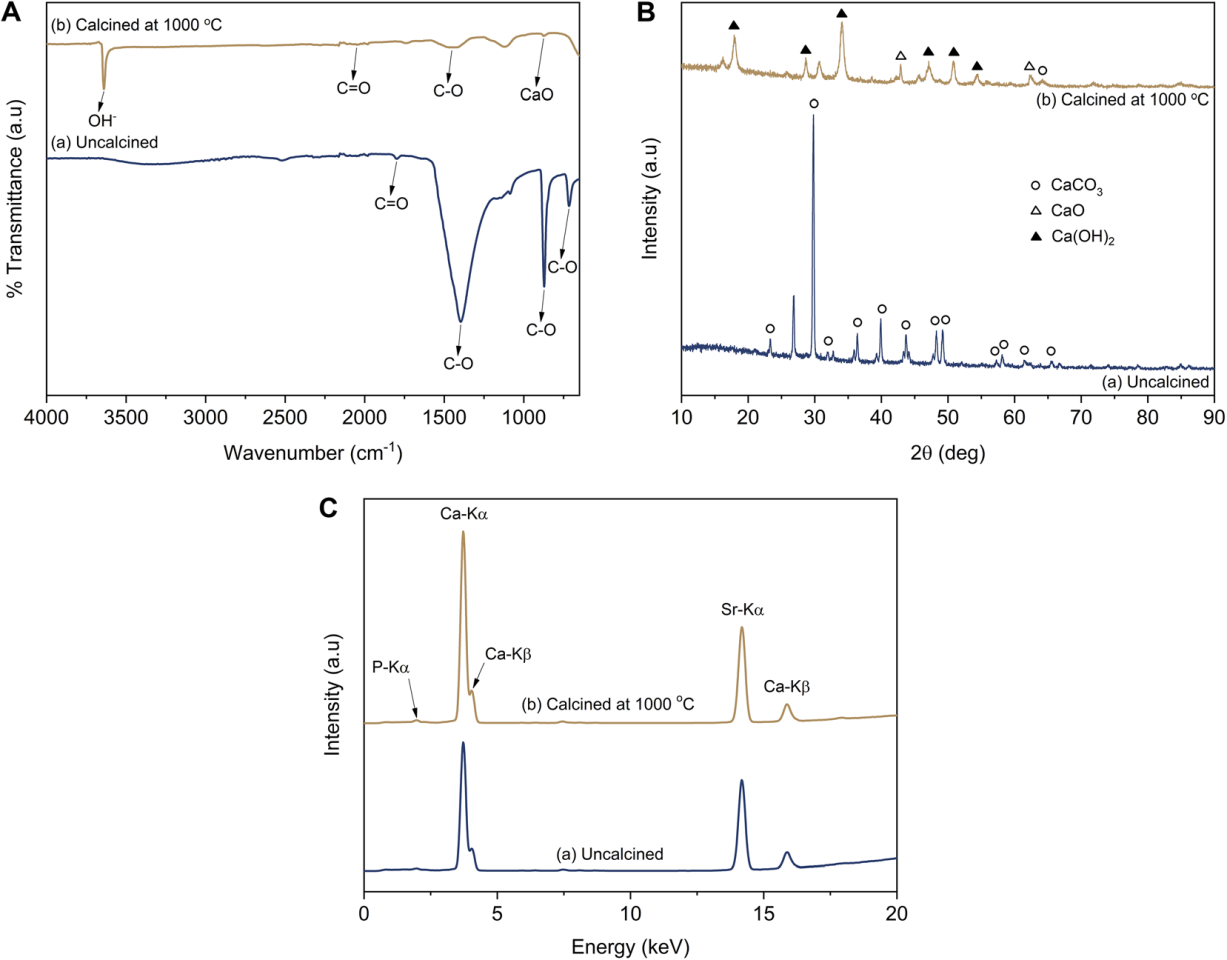
(A) Infrared spectra, (B) diffraction pattern, and (C) fluorescence spectra of the LSSU shells.

According to the results from the infrared spectral analysis, the infrared spectra of the uncalcined LSSU shells showed C–O and C=O bonds, which were identified as CaCO_3_ functional groups (Fig. 2A). The C–O bond functional groups were observed at 1396 cm^−1^, 872 cm^−1^, and 716 cm^−1^, whereas the C=O bond functional group was observed at 1799 cm^−1^. After the LSSU shells were calcined at 1000 °C, the CaO bond functional group occurred at 875 cm^−1^, followed by very drastic decrease in the C–O bond peak, which indicated that there had been a decomposition process to break the chemical bond of the CaCO_3_ mineral into CaO and this had released CO_2_. However, the OH^−^ stretching functional group which appeared at 3639 cm^−1^ was due to the phase change from CaO into Ca(OH)_2_, and was possibly caused by the high reactivity of CaO interacting with H_2_O.^36^ Another C=O bond functional group was also observed at 2050 cm^−1^ in the calcined LSSU shells.

As shown in Fig. 2B, the diffraction pattern analysis of the uncalcined LSSU shells showed diffraction peaks corresponding to CaCO_3_ (calcite, PDF number 47-1743), which were strongly shown at a diffraction angle (2*θ*) of 29.76°. The LSSU shells had a crystallite size and a microstrain of 31.83 ± 4.71 nm and 0.0228, respectively, with lattice parameters of *a* = 4.9347 Å and *c* = 16.8226 Å. In comparison, the calcined LSSU shells showed the presence of Ca(OH)_2_ (portlandite, PDF number 44-1481) with a low content of CaO (lime, PDF number 37-1497). Their crystallite sizes were 12.32 ± 2.86 and 9.62 nm, whereas their microstrains were 0.0727 and 0.1096, respectively. The Ca(OH)_2_ had lattice parameters of *a* = 3.5915 Å and *c* = 4.9353 Å with a hexagonal structure, whereas the CaO had a lattice parameter of *a* = 4.8125 Å with a cubic structure. A large Ca(OH)_2_ crystal formation was confirmed, and with the amorphous phase level increase, this was due to the very small amount of pure CaO present before the characterisation. The hygroscopic properties of the LSSU shells caused them to absorb H_2_O and significantly decreased the CaO crystal obtained after calcination.

Based on the fluorescence spectral analysis, calcined LSSU shells showed a higher Ca content, which was followed by a higher intensity of Ca-Kα and Ca-Kβ (Fig. 2C). This was confirmed by the Ca level revealed in Table 1, which was about 94.70% in uncalcined LSSU shells, whereas in calcined LSSU shells it was approximately 96.06%. The increase in Ca level referred to the calcination temperature effect that broke the chemical bond of calcite and released CO_2_. Phosphor (P), strontium (Sr), and other inorganic elements were observed as impurities but remained at low levels.

**Table 1:** Atomic levels of LSSU shells

No.	Element	Element level (%)	
		Uncalcined	Calcined at 1000 °C
1	Ca	94.70	96.06
2	P	2.22	1.72
3	Sr	1.28	0.96
4	Other elements	1.80	1.26

### 3.2. Properties of the HAp and C-HAp nanorods based on LSSU shells

**Fig. 3:**
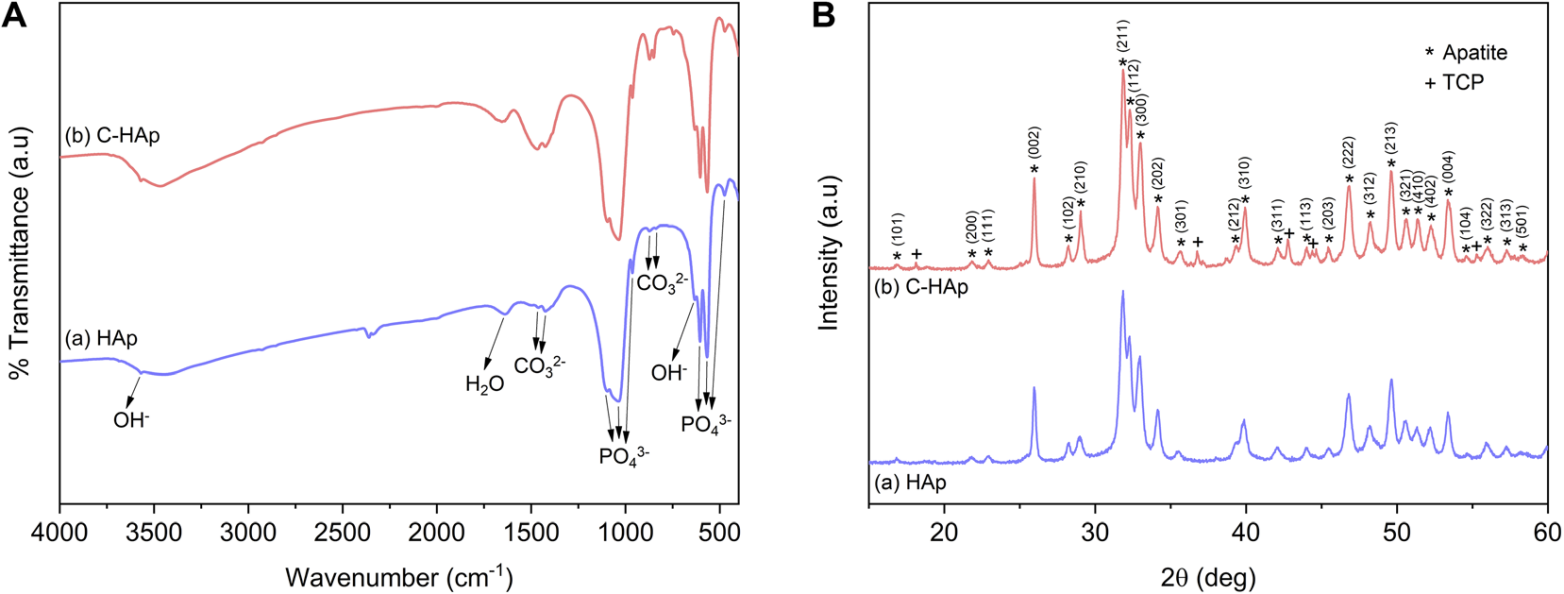
(A) Infrared spectra, and (B) diffraction pattern of the HAp and C-HAp nanorods.

The infrared spectra of the HAp and C-HAp nanorods are shown in Fig. 3A, and they have similar patterns. The peaks at 475, 567–605, 963, and 1038–1096 cm^−1^ were attributed to the stretching mode of *ν*_2_ PO_4_^3−^, the asymmetric bending mode of *ν*_4_ PO_4_^3−^, the stretching mode of *ν*_1_ PO_4_^3−^, and the asymmetric stretching mode of *ν*_3_ PO_4_^3−^, respectively. The small peaks at 632 and 3569 cm^−1^ corresponded to the bending and stretching modes of OH^−^. The absorption band of the absorbed water appeared at 1639 cm^−1^. The HAp showed a slight peak of carbonate ions presented at the *ν*_2_ and *ν*_3_ regions due to CaO interaction with Co_2_ in free air during HAp synthesis before the hydrothermal reaction.^15^ In the synthesised C-HAp, the presence of the stretching mode of *ν*_3_ CO_3_^2−^ at 1467 and 1425 cm^−1^ indicated carbonated hydroxyapatite (CHAp) type AB within the A-site (OH site), and the B-site (PO_4_ site), respectively. The typical CHAp type AB corresponded to the stretching mode of *ν*_2_ CO_3_^2−^ which were also found at 851 and 873 cm^−1^.

The diffraction pattern of HAp and C-HAp showed observable peaks which corresponded to apatite peaks (PDF number 09-0432) (Fig. 3B). The main characteristic peaks formed at the diffraction angle (2*θ*) of 31.9°, 32.3°, 33°, and 34.1° were attributed to the crystal planes of (211), (112), (300), and (202), respectively. The effect of the carbonate ion substitution on the HAp crystal structure was seemingly insignificant according to C-HAp diffraction peaks. However, the high temperature of the dissolution–precipitation process caused the appearance of tricalcium phosphate (TCP) within α and β-type with low intensity. As a tertiary calcium phosphate, TCP is extremely biocompatible for biological hard tissue improvement.^52^ The substitution of a carbonate ion into the HA lattice structure reduced the lattice parameter of *a* and increased the lattice parameter of *c* (Table 2). Based on the FTIR data, the carbonate ion tended to be substituted for hydroxyl or phosphate ions, which caused a decrease in the crystallinity. The lower crystallinity of the C-HAp nanorods caused it to be more soluble in biological conditions, which is good for cell growth. The substituted phosphate ion in C-HAp was indicated by the Ca/P value of C-HAp, which was higher than that of HAp (Table 2). The Ca/P of C-HAp was 1.705, which was very close to the apatite structure in bone tissue at 1.71.

**Table 2:** Physicochemical properties of HAp and C-HAp nanorods

No.	Sample	Particle size (nm)		Ca/P	Lattice parameters			Degree of crystallinity (%)
		Diameter	Length		*a* (Å)	*c* (Å)	*c*/*a*	
1	HAp	18.57 ± 1.85	57.80 ± 2.93	1.669	9.4113	6.8568	0.729	78.2
2	C-HAp	16.14 ± 1.86	121.48 ± 3.45	1.705	9.4014	6.8595	0.730	77.9

**Fig. 4:**
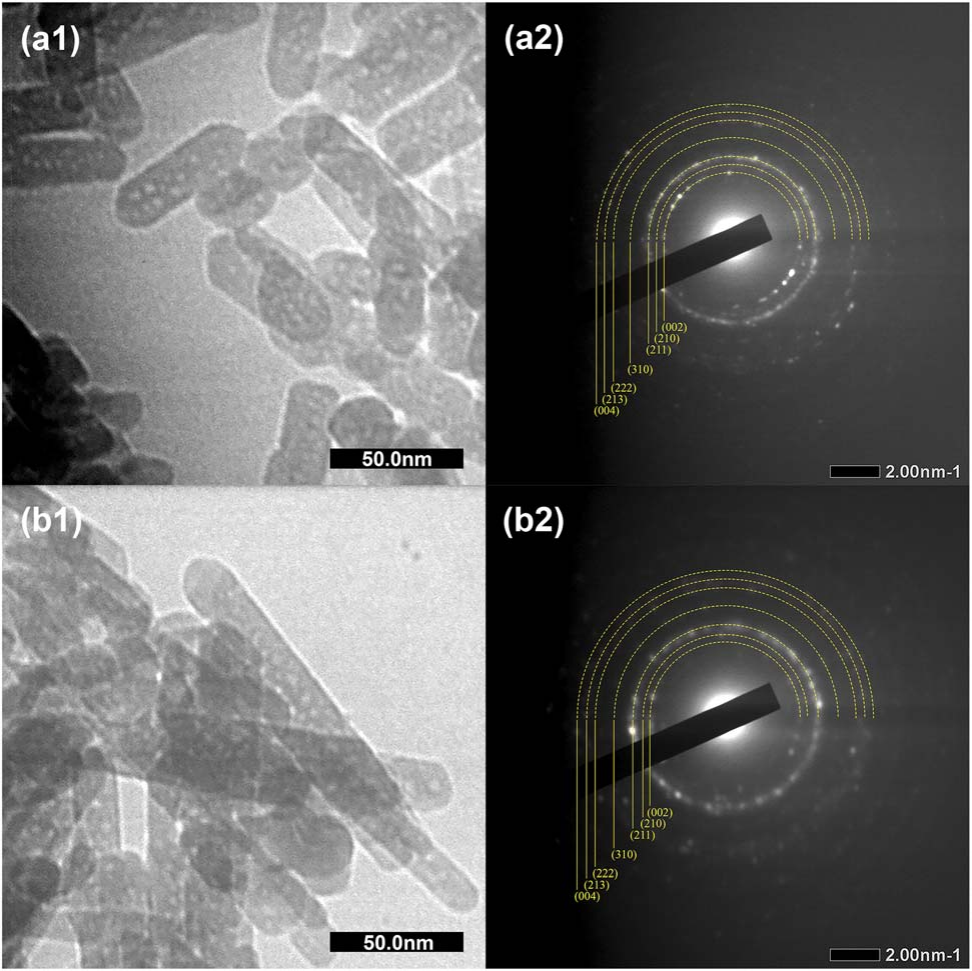
The TEM micrographs and SAED patterns of (a1 and a2) Hap, and (b1 and b2) C-HAp nanorods.

Fig. 4 shows the morphology of the HAp and C-HAp nanoparticles, including the selected area electron diffraction (SAED) patterns. Both HAp and C-HAp had a nanosized rod-like shape. According to Table 2, the C-HAp had longer length and a smaller diameter than HAp. The higher aspect ratio of C-HAp provided a larger surface area, promoting ion exchange and cell interactions.^35^ The SAED pattern of HAp and C-HAp showed the hkl lattice spacing which was ascribed to the lattice plane of the hexagonal crystalline structure of the apatite. These diffraction ring patterns validated the XRD data.

### 3.3. Properties of CNF

**Fig. 5:**
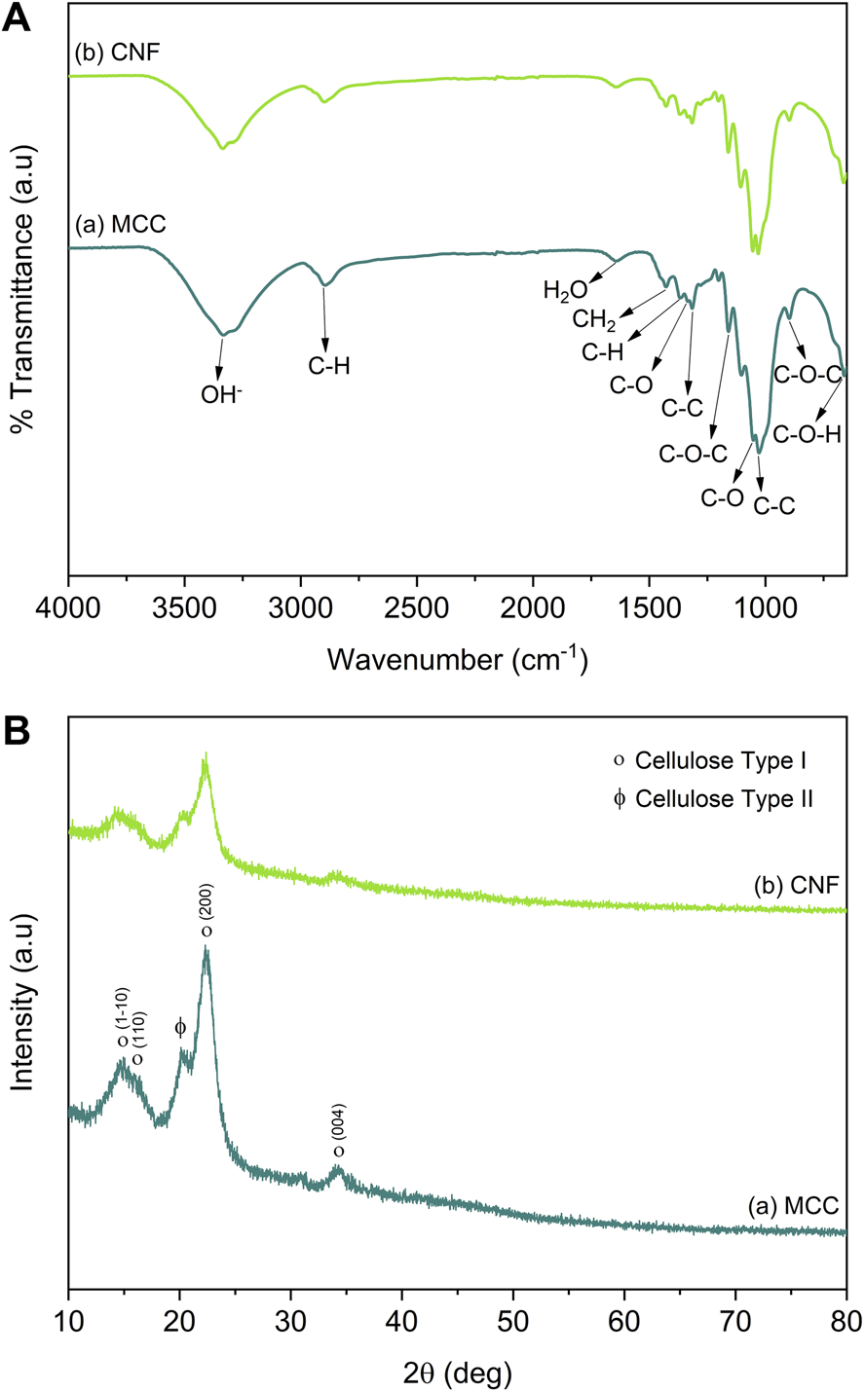
(A) Infrared spectra, and (B) the diffraction patterns of MCC and CNF.

The MCC and CNF showed very similar infrared spectra (Fig. 5A). The broad peak at 3332–3335 cm^−1^ was attributed to the stretching mode of OH^−^. The OH^−^ stretching in the CNF had a sharper peak when compared to that of the MCC, which indicated that the increase of the hydrogen bonding facilitated re-aggregation of nanocellulose as a consequence of the ultrasonication process.^53^ The peaks at 1367–1368 and 2896–2899 cm^−1^ were attributed to the symmetric bending and stretching modes of C–H, respectively. The absorption band at 1644 cm^−1^ corresponded to the bending mode of the absorbed water. The band at 1428 cm^−1^ was assigned to the symmetric bending mode of CH_2_. The C–O–C stretching vibration at the β-(1,4)-glycosidic linkages appeared at 1159–1160 and 898 cm^−1^. The C–C and C–O skeletal vibrations appeared at 1315 and 1335 cm^−1^. Meanwhile, the strong peaks of C–C stretching and C–O stretching at the C_3_ position appeared at 1028–1032 and 1052–1055 cm^−1^, respectively. The small peak at 662–663 cm^−1^ was related to the bending mode of C–O–H. The spectra of MCC and CNF had no significant differences, and it was concluded that the ultrasonication treatment did not break the basic chemical structure of MCC, and no derivational reaction occurred.^54^

**Fig. 6:**
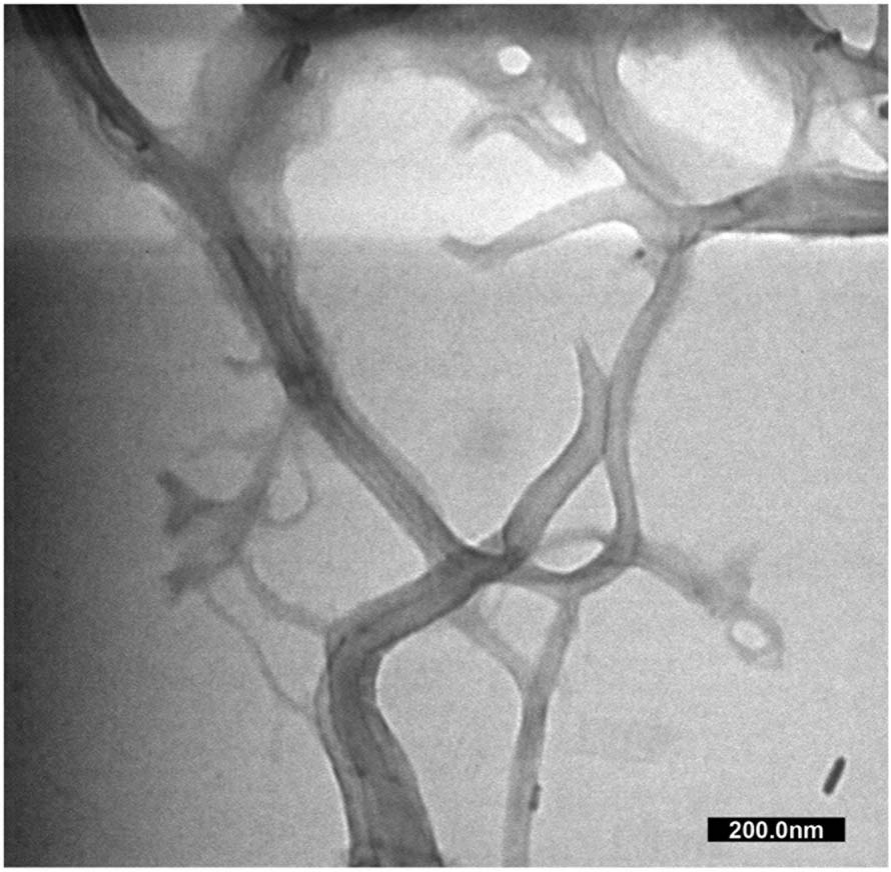
The TEM micrograph of CNF.

In Fig. 5B, the diffraction pattern of MCC and CNF showed significant changes in peak intensity. The characteristic peaks referred to as cellulose type I are shown at the diffraction angle (2*θ*) of 14.7°, 15.9°, 22.4°, and 34.3°, corresponding to the crystal planes of (1–10), (110), (200), and (004), respectively.^55^ These main diffraction peaks were consistent with the FTIR results, but a small peak at 20.4° beside the (200) crystal plane appeared and was related to the cellulose type II.^49^ The CNF decreased the peak intensity of the MCC, indicating that the lower crystallinity was due to the ultrasonication. The cavitation effect in the ultrasonic treatment destroyed the cellulose crystalline phase, reducing the peaks' intensity and resulting in nanofibrillated cellulose.^56^ The nanofibrillated morphology of CNF is identified in Fig. 6. The CNF showed a curled and straightened fibril surrounded with a gel-like structure resulting from the highly entangled fibril. From the micrograph, the nanofibril diameters varied from 10 to 100 nm. The CNF showed a high aspect ratio, but it could not be determined accurately.

### 3.4. Properties of the C-HAp/PVA-based scaffolds

#### 3.4.1. Physicochemical characteristics of the C-HAp/PVA-based scaffolds

**Fig. 7:**
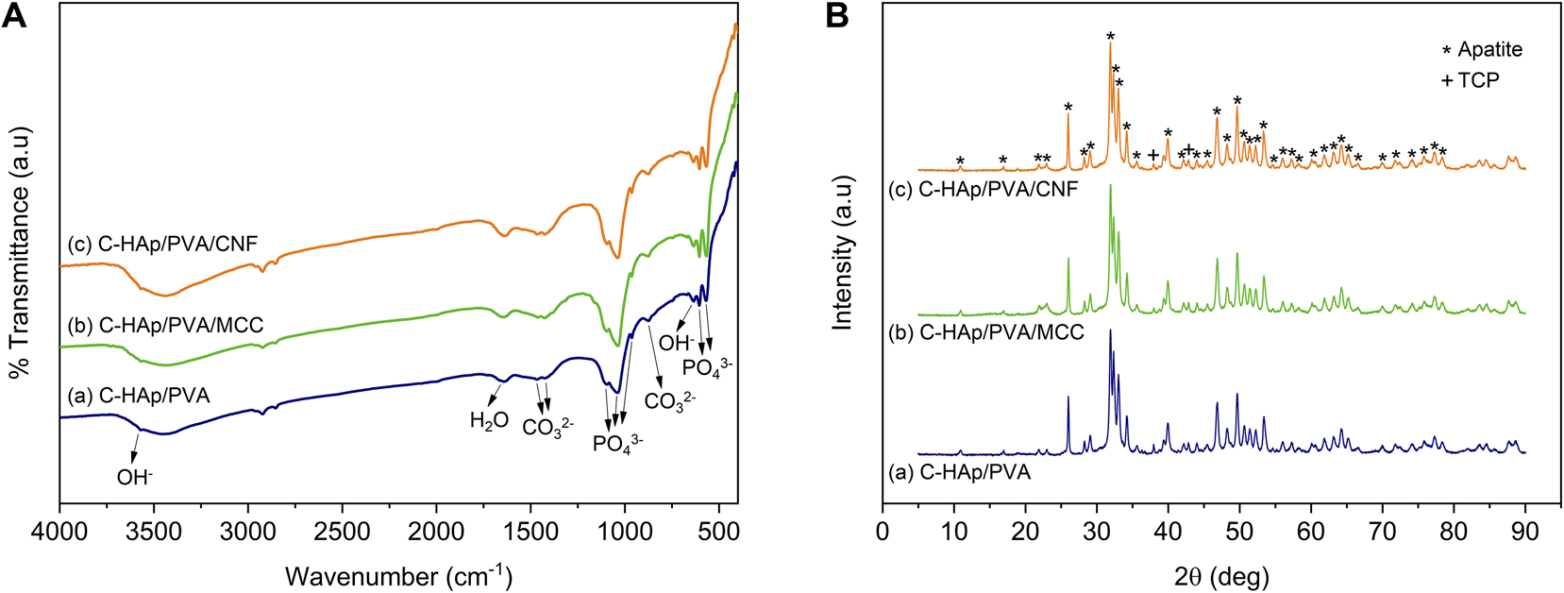
(A) Infrared spectra, and (B) diffraction patterns of the C-HAp/PVA-based scaffolds.

The infrared spectra of the scaffolds are shown in Fig. 7A. The addition of a low concentration of PVA caused the main peaks of C-HAp to become weaker. However, the peaks at 2924 and 2854 cm^−1^ became stronger, which was attributed to C–H stretching, which indicated the presence of PVA as a polymeric reinforcement. The addition of MCC and CNF did not lead to significant differences in the C-HAp and PVA-based scaffold. In all scaffolds, the peaks displayed at 568–605, 963–964, and 1036–1095 cm^−1^ corresponded to the vibration of *ν*_4_ PO_4_^3−^ asymmetric bending, the *ν*_1_ PO_4_^3−^ stretching, and the *ν*_3_ PO_4_^3−^ asymmetric stretching, respectively. The small peaks at 635 and 3568–3570 cm^−1^ were related to the OH^−^ bending and stretching vibrations. The absorption band at 1640–1643 cm^−1^ was attributed to absorbed water. No increase occurred in the peak of the carbonate group at 1426–1468 and 876–878 cm^−1^ because the lyophilisation of the scaffold involved a low temperature. The infrared spectra graph of the C-HAp-based scaffolds showed no decomposition of the chemical structure of apatite.

The diffraction analysis showed that the scaffolds strongly exhibited their apatite crystal because of the low fraction of polymer and cellulose in the scaffold structure (Fig. 7B). However, significant effects occurred in degree of crystallinity (Table 3). The amorphous polymeric PVA caused the C-HAp/PVA scaffold to have a lower crystallinity. The addition of MCC and CNF also reduced the crystallinity of the scaffold. The low crystallinity encourages biodegradability of the scaffold together with the growth of new bone tissue. The lower crystallinity indicated dislocation in the scaffold structure, which can help cells proliferate more easily. Hence, crystallinity can influence the ability of the cells to attach and differentiate on the scaffold surface.^37^

**Table 3:** Crystallinity of the C-HAp/PVA-based scaffolds

No.	Sample	Degree of crystallinity (%)
1	C-HAp	78.2
2	C-HAp/PVA	77.0
3	C-HAp/PVA/MCC	76.5
4	C-HAp/PVA/CNF	75.3

**Fig. 8:**
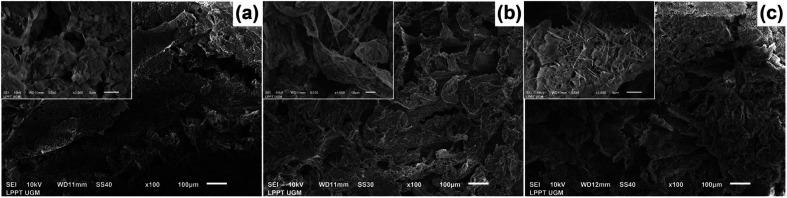
The morphology of (a) C-HAp/PVA, (b) C-HAp/PVA/MCC, and (c) C-HAp/PVA/CNF.

The morphology of all the scaffolds had a porous structure with a heterogeneous macropore structure (Fig. 8). The C-HAp/PVA scaffold had a small number of macropores with a size of less than 100 μm (Fig. 8a). As shown in Fig. 8(b) and (c), adding MCC and CNF into the scaffold decreased the size of the macropores which become even smaller with a heterogeneous distribution. Interestingly, the C-HAp/PVA/CNF had a lamellar structure which was visible from its surface, and this allowed cell distribution and blood vessel formation in the lamellae interstices.^34^ However, the C-HAp/PVA/MCC had a more dense macrostructure due to its large and randomly distributed micro-cellulose particles that tended to make bigger pores, and consequently, it had no micropores (in the microscale image). Although the C-HAp/PVA had a few micropores, their distribution was still uneven. The C-HAp/PVA/CNF showed a greater micropore distribution with a smaller micropore size, causing it to be more suitable for cell migration.

#### 3.4.2. Antibacterial activity of C-HAp/PVA-based scaffolds

**Fig. 9:**
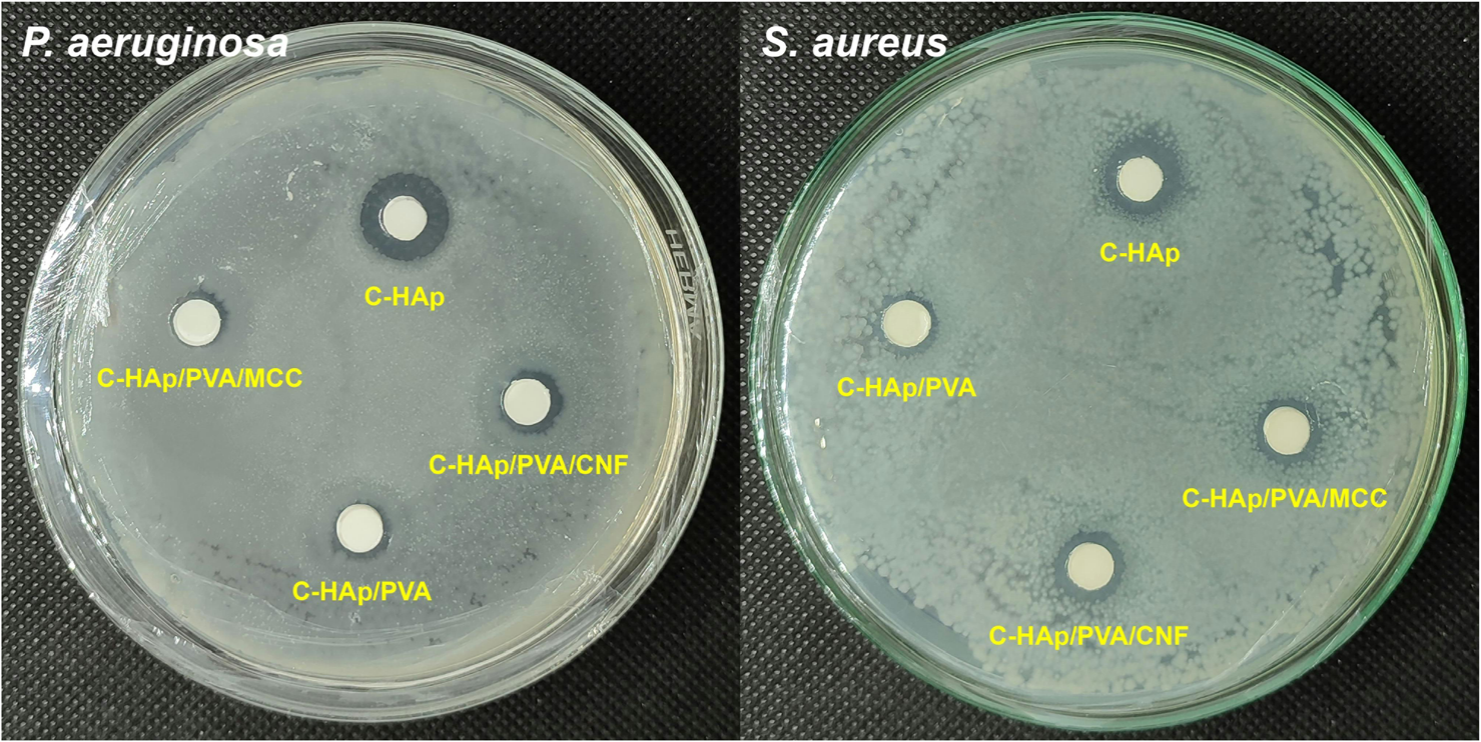
The antibacterial activity of the C-HAp nanorods and the C-HAp/PVA-based scaffolds.

**Fig. 10:**
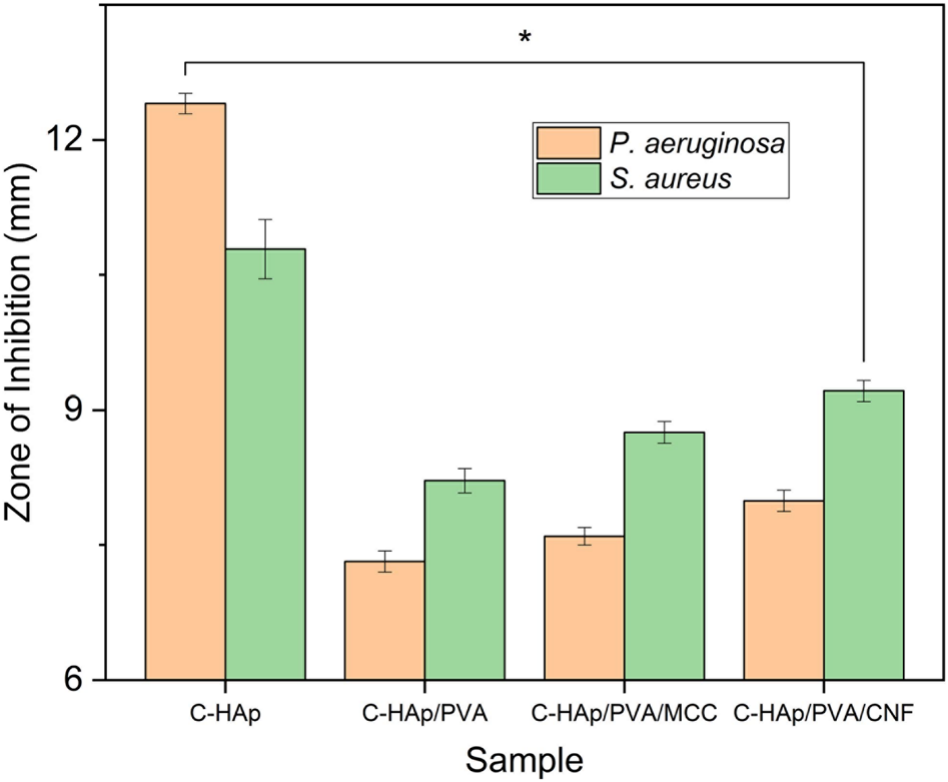
The ZOI values (*p-value < 0.05) of the C-HAp nanorods and C-HAp/PVA-based scaffolds.

The C-HAp/PVA-based scaffolds and the C-HAp nanorods exhibited antibacterial ability against *S. aureus* and *P. aeruginosa* (Fig. 9), and the diameters of the ZOI formed are included in Table 4. According to the Bauer–Kirby disc diffusion theory, the value of the ZOI based on diameter includes medium (5–10 mm), strong (10–20 mm), and very strong (>20 mm).^57^ The ZOI value of the C-HAp nanorods was relatively strong, so the C-HAp nanorods had a strong inhibition against the two bacteria (*P. aeruginosa* and *S. aureus*) that cause periodontitis. The reinforcement of the scaffold with PVA decreased the antibacterial activity of C-HAp because the PVA had no direct bacterial inhibition (Fig. 10). The ZOI value of the C-HAp/PVA scaffold decreased to relatively medium, which means that the scaffold had a medium inhibition against the bacteria. The addition of MCC into the scaffold increased the diameter of the ZOI. The antibacterial properties of MCC have been investigated in previous research, and the MCC had a medium inhibition against bacteria such as *S. aureus*.^58^ Furthermore, the addition of CNF into the scaffold could enhance the antibacterial activity more than MCC could. The antibacterial properties of CNF were induced by multiple reactive hydroxyl groups on the surface of CNF, which could react with the aminoalkyl groups and lead to lethal activity against bacteria.^59,60^ Because the CNF had a larger surface area than MCC, the CNF contained more reactive-hydroxyl groups and induced a higher bacteriostatic effect. The increase of the hydroxyl groups was confirmed using the FTIR data. Although the ZOI value of the scaffolds remained medium, the scaffolds have potential for inhibiting bacterial growth.

**Table 4:** The ZOI values of C-HAp/PVA-based scaffolds

No.	Sample	Diameter of ZOI (mm)	
		*P. aeruginosa*	*S. aureus*
1	C-HAp	12.40 ± 0.11	10.79 ± 0.33
2	C-HAp/PVA	7.32 ± 0.12	8.21 ± 0.13
3	C-HAp/PVA/MCC	7.59 ± 0.10	8.75 ± 0.12
4	C-HAp/PVA/CNF	7.99 ± 0.12	9.21 ± 0.12

#### 3.4.3. The micromorphology of the C-HAp/PVA/CNF scaffold

**Fig. 11:**
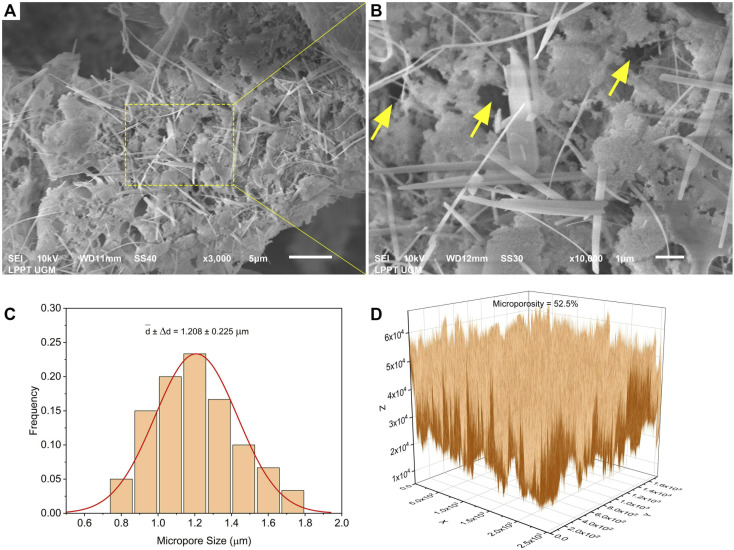
(A) The micromorphology, (B) the magnified micromorphology, (C) the average micropore sizes, and (D) the microporosity of the C-HAp/PVA/CNF scaffold.

Based on the SEM results shown in Fig. 11A, the microscale morphology of the C-HAp/PVA/CNF scaffold shows the structure of the micropores. The micropores are marked with yellow arrows (Fig. 11B). The micropores were formed by ice crystals that were sublimated during lyophilisation and left the densification of the apatite nanoparticles and the cellulose nanofibrils reinforced with polymeric PVA. The nanosize of the cellulose fibrils allowed them to be distributed evenly around the C-HAp nanorods. The distribution of the micropore sizes is displayed in Fig. 11C. However, the sizes of the micropores were distributed heterogeneously due to inhomogeneous suspension of the scaffold before it was stored in the deep freezer, thus creating heterogeneous pore sizes with random interconnectivity. According to ImageJ measurements, the average micropore size was 1.208 ± 0.225 μm. This micropore size was below 20 μm, which can be regarded as medium for cellular growth.^61^ The two-dimensional microporosity analysis of the scaffold surface was conducted using Origin software (OriginLab, Northampton, MA, USA) that transformed the SEM micrograph into a 3D graph of micropore distribution. The microporosity was analysed using a calculation of solid volume, integral volume, and micropore volume on the 3D graph. The micropore distributions among the solid particles were evaluated using colour distribution. In Fig. 11D, a light colour represented solid particles, whereas a dark colour represented micropores. According to the measurements, the microporosity of the scaffold was 52.5%. This value was closely related to the micropore size used to promote nutrient supply for bone tissue.^62^ Therefore, the C-HAp/PVA/CNF scaffold had optimal micropore sizes and microporosity, so it has potential for use in hard tissue regeneration.

#### 3.4.4. Cell viability of the C-HAp/PVA/CNF scaffold

**Fig. 12:**
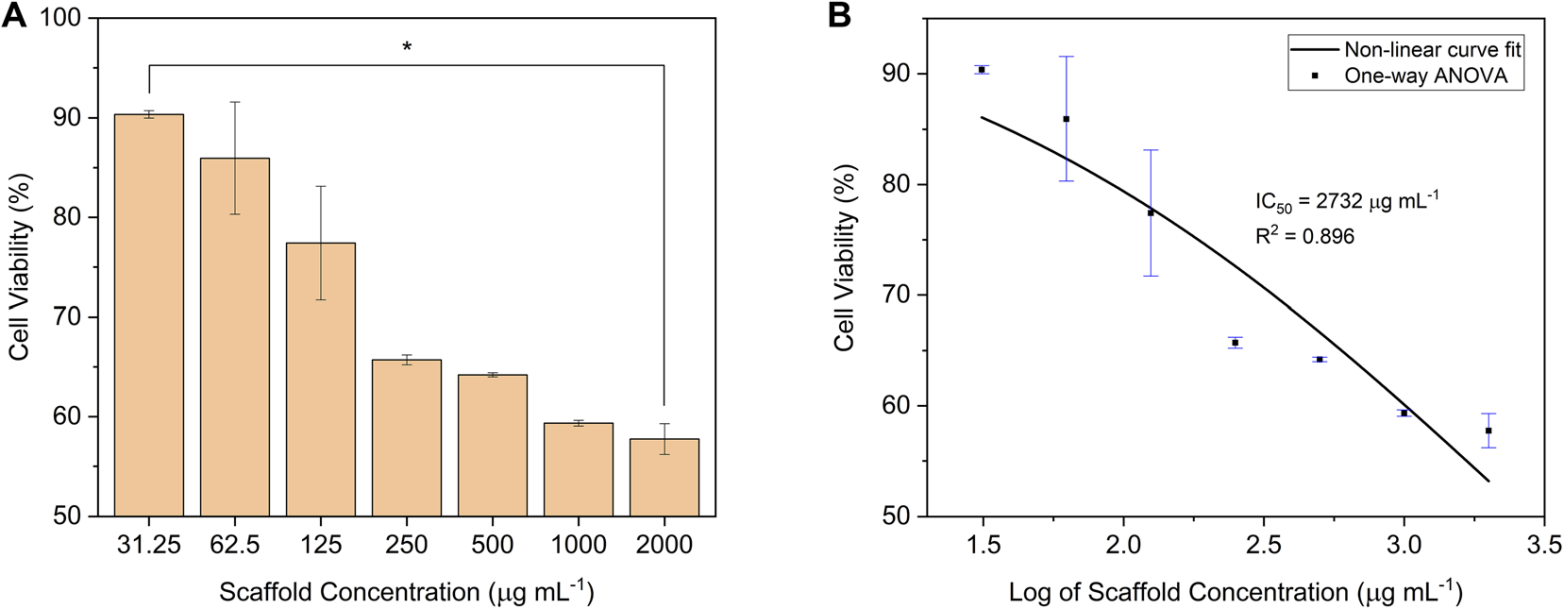
(A) The cell viability (*p-value < 0.05), and (B) the IC_50_ of the C-HAp/PVA/CNF scaffold after 24 h of incubation against NIH/3T3 fibroblast cells.

The MTT assay was performed to determine the cytocompatibility of the C-HAp/PVA/CNF scaffold against the NIH/3T3 fibroblast cells. After 24 h incubation, the scaffold showed a high cell viability at a lower scaffold concentration, and it was assessed to be safe (Fig. 12A). According to data given in Table 5, the cell viability of the scaffold was 90.36% at a scaffold concentration of 31.25 μg mL^−1^. The increase in the scaffold concentration caused the cell viability to decrease. Based on the one-way ANOVA statistical analysis, the *p*-value was less than 0.05, meaning the serial scaffold concentration had significantly affected the cell viability value. At higher scaffold concentrations, the viability of the NIH/3T3 cells was close to the cell viability at the IC_50_ value. In Fig. 12B, the IC_50_ value was calculated from the statistical data obtained using non-linear curve fitting, and it was estimated at a scaffold concentration of 2732 μg mL^−1^. At this IC_50_ value, the NIH/3T3 cells were considered to have the maximum safe dose. However, the viability assay showed that the C-HAp/PVA/CNF had a cytocompatible interaction with the NIH/3T3 cells.

**Table 5:** Average cell viability of the C-HAp/PVA/CNF scaffold

No.	Scaffold concentration (μg mL^−1^)	Cell viability (%)	*p*-Value
1	31.25	90.36 ± 0.37	*p* < 0.05
2	62.5	85.94 ± 5.62	
3	125	77.42 ± 5.71	
4	250	65.71 ± 0.50	
5	500	64.20 ± 0.20	
6	1000	59.34 ± 0.29	
7	2000	57.75 ± 1.55	

**Fig. 13:**
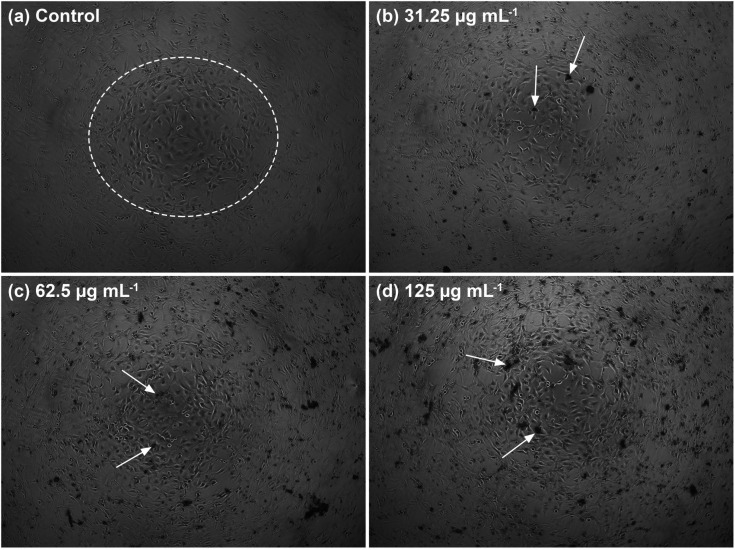
The morphology of the NIH/3T3 fibroblast cells after 24 h incubation in low serial scaffold concentrations: (a) control, (b) 31.25, (c) 62.5, and (d) 125 μg mL^−1^.

The morphology of the well-connected NIH/3T3 cells in the cell network is shown in Fig. 13a. In the white circle, the cells formed several sub-confluent structures to 80% and were mostly clustered together. The morphology showed that the C-HAp/PVA/CNF scaffold was attached to the cells after 24 h incubation (Fig. 13(b)–(d)). The dark black colour around the NIH/3T3 cells referred to the scaffold. According to the micrographs, the scaffold surface promoted attachment of the NIH/3T3 cells, indicated by the white arrows. The scaffold contained the C-HAp nanorods with high osteoconductivity resulting from its physicochemical properties that encouraged the cells to attach to the scaffold surface.^63^ The biocompatibility of the CNF also triggered cell activity because the higher hydroxyl content on the CNF surface could interact with water and enhanced the cabality for cell attachment.

## 4. Conclusion

This study presents the synthesis of C-HAp using the dissolution–precipitation of HAp. The HAp nanorods were prepared using a hydrothermal method based on calcined LSSU shells at 1000 °C. The synthesized C-HAp achieved a molar ratio of Ca/P = 1.705, which is very close to the Ca/P of biological apatite of 1.71. The C-HAp nanorods formed CHAp type AB. According to FTIR, XRD, and TEM results, the C-HAp nanorods had better physicochemical properties and were more suitable for scaffold fabrication for alveolar bone tissue engineering. The C-HAp-based scaffolds were fabricated using PVA as a reinforcing polymer, with various additions of cellulose material, including MCC and CNF. The CNF was prepared from MCC using ultrasonic treatment. The CNF achieved a high aspect ratio and a large nanosized surface area with good chemical characteristics. Based on the FTIR and XRD results, the C-HAp-based scaffolds showed no decomposition of the chemical structure of apatite. However, the degree of crystallinity decreased with the addition of polymer and cellulose. The C-HAp/PVA/CNF scaffold was considered for a viability assay because its lowest crystallinity was better for bone growth and helped cells to proliferate easily. The antibacterial test result also showed that the C-HAp/PVA/CNF had a higher antibacterial activity than the other scaffolds. From the SEM results, the C-HAp/PVA/CNF exhibited the correct macropore size and microporosity, which could encourage cellular growth and nutrient supply for the cells. It also had appropriately sized macropores for cell distribution and blood vessel formation. The cytocompatibility of the C-HAp/PVA/CNF scaffold was validated using the MTT assay results, which showed the high cell viability of the NIH/3T3 fibroblast cells at lower scaffold concentrations and facilitated the attachment of the NIH/3T3 cells to the scaffold surface.

## Conflicts of interest

The authors declare no conflicts of interest.

## Supplementary Material
