# Correction: Functionalized cellulose nanofibrils in carbonate-substituted hydroxyapatite nanorod-based scaffold from long-spined sea urchin (*Diadema setosum*) shells reinforced with polyvinyl alcohol for alveolar bone tissue engineering

**DOI:** 10.1039/d3ra90115g

**Published:** 2023-12-04

**Authors:** Muhammad Amir Jamilludin, I Kadek Hariscandra Dinatha, Apri I Supii, Juliasih Partini, Dwi Liliek Kusindarta, Yusril Yusuf

**Affiliations:** a Department of Physics, Faculty of Mathematics and Natural Sciences, Universitas Gadjah Mada Yogyakarta 55281 Indonesia yusril@ugm.ac.id; b Research Centre for Marine and Land Bioindustry, National Research and Innovation Agency Lombok Utara 83352 Indonesia; c Department of Anatomy, Faculty of Veterinary Medicine, Universitas Gadjah Mada Yogyakarta 55281 Indonesia

## Abstract

Correction for ‘Functionalized cellulose nanofibrils in carbonate-substituted hydroxyapatite nanorod-based scaffold from long-spined sea urchin (*Diadema setosum*) shells reinforced with polyvinyl alcohol for alveolar bone tissue engineering’ by Muhammad Amir Jamilludin *et al.*, *RSC Adv.*, 2023, **13**, 32444–32456, https://doi.org/10.1039/D3RA06165E.

The authors regret that the C-HAp degree of crystallinity is incorrectly presented in Table 3 in the original article. The corrected version of Table 3 is shown below.

**Table:** Crystallinity of the C-HAp/PVA-based scaffolds

No.	Sample	Degree of crystallinity (%)
1	C-HAp	77.9
2	C-HAp/PVA	77.0
3	C-HAp/PVA/MCC	76.5
4	C-HAp/PVA/CNF	75.3

The Royal Society of Chemistry apologises for these errors and any consequent inconvenience to authors and readers.

## Supplementary Material

